# Erectile function, urinary continence and oncologic outcomes of neurovascular bundle sparing robot-assisted radical prostatectomy for high-risk prostate cancer: A systematic review and meta-analysis

**DOI:** 10.3389/fonc.2023.1161544

**Published:** 2023-04-05

**Authors:** Yang Liu, Xian-zhong Deng, Jiao Qin, Zhi Wen, Yu Jiang, Jing Huang, Chong-jian Wang, Cai-xia Chen, Li Wang, Kun-peng Li, Jia-hao Wang, Xue-song Yang

**Affiliations:** ^1^ Department of Urology, Affiliated Hospital of North Sichuan Medical College, Nanchong, China; ^2^ Department of Urology, Chengdu Xinhua Hospital Affiliated to North Sichuan Medical College, Chengdu, China; ^3^ Department of Anesthesia, Affiliated Hospital of North Sichuan Medical College, Nanchong, China; ^4^ Department of Radiate, Affiliated Hospital of North Sichuan Medical College, Nanchong, China; ^5^ Department of Urology, Lanzhou University Second Hospital, Lanzhou, China

**Keywords:** high-risk prostate cancer, robot-assisted radical prostatectomy, neurovascular bundle, outcomes, continence, meta-analysis

## Abstract

**Background:**

The nerve-sparing (NS) effect of robot-assisted radical prostatectomy (RARP) on patients with a high-risk prostate cancer remains unclear. The objective of this study was to compare the urinary continence, erectile function and oncology outcomes of the nerve-sparing and non-nerve-sparing (NNS) group during RARP surgeries.

**Methods:**

We systematically searched databases including PubMed, Embase, Cochrane Library and Web of Science to identify relevant studies published in English up to December 2022. Newcastle-Ottawa Scale (NOS) was used as a quality evaluation tool to evaluate the quality of the literature parameters involved, including urinary continence, erectile function and oncologic outcomes, which were compared using the Stata 15.1 software (StataSE, USA).

**Results:**

A total of 8 cohort studies involving 2499 patients were included. A meta-analysis of results showed that the NS group was beneficial to the recovery of urinary continence (RR 0.46, 95%CI 0.22, 0.96; p=0.045<0.05) and erectile function (RR 0.32, 95%CI 0.16, 0.63; p=0.001<0.05) 12 months after surgeries, which showed a better oncological outcome (RR 1.31, 95%CI 1.01, 1.69; p=0.01<0.05).

**Conclusions:**

The current study results indicate that intraoperative NS during RARP is beneficial to long-term postoperative functional recovery and tumor prognosis of patients with high-risk prostate cancers. Due to interstudy interferences, the results should be interpreted with caution.

**Systematic review registration:**

https://www.crd.york.ac.uk/PROSPERO/, identifier: CRD42022384647.

## Introduction

Prostate cancer (Pca) is the second most common solid tumor for men (1). In the United States, 191,930 new cases with Pca are expected in 2020 (2). Although the mortality of prostate has been stabilized in developed countries in recent years, it is still the leading cause of deaths for male patients with cancers (3, 4). High-risk prostate cancer was defined with a PSA≥20ng/ml, a Gleason score of 8-10 or a clinical stage ≥T3, which accounted for about 40% of the total (5). Robot-assisted radical prostatectomy (RARP) is currently a common method for treating localized Pca (6), which provides a higher field of view and more freedom compared with traditional radical prostatectomy (RP). These advantages make intraoperative NS more feasible. The perioperative and functional outcomes of the treatment of localized Pca through robot-assisted, laparoscopic or open radical prostatectomy differ (7).

Nerve sparing (NS) is a topic well worth exploring in the surgical management of patients with high-risk Pca, and many studies have confirmed that NS helps to improve the functional prognosis of patients in radical prostatectomy (8–10). Several meta-analyses have demonstrated that NS is beneficial to the postoperative recovery of urinary continence and erectile function (EF), which reduces the risk of postoperative urinary incontinence (11–13). Veneziano et al. (14) suggested that NS was beneficial for patients with urinary incontinence, which significantly improved the EF of high-risk patients. The effect of NS selection on patients in RARP is still unclear, so further exploration is very necessary.

Therefore, we conducted a meta-analysis of the published clinical studies on the outcomes of NS in RARP to provide the latest evidence for clinicians to make decisions.

## Methods

We conducted a systematic review and meta-analysis following the guideline of the *Preferred Reporting Items for Systematic Reviews and Meta-Analyses* (PRISMA) (15). A relevant protocol has been registered on the Prospero website (https://www.crd.york.ac.uk/PROSPERO/) with a registration number of CRD42022384647.

### Literature search strategy, study selection and data collection

The databases Pubmed, Cochrane Library, Web of Science and Embase were searched, and the deadline for literature search was December 8, 2022. According to the PICOS standard definition of search terms, literature search was conducted by combining the topic with free terms: (((high-risk) AND ((prostate cancer) OR (prostate neoplasm))) AND ((NS) OR (neurovascular bundle sparing))) AND ((Robotic surgical procedures) OR (RARP)).

The literature to be searched was written in English language only, so we also manually searched and reviewed the relevant references to avoid any omission.

We used the PICOS approach to define the inclusion criteria. P (patients): all the patients were diagnosed with a high-risk prostate cancer (PSA≥20ng/ml or a Gleason score of 8-10 or a clinical stage ≥T3) and undergone RARP; I (intervention): NS, bilateral nerve-sparing (BNS), unilateral nerve-sparing (UNS) or partial nerve-sparing (PNS); C (comparator): non-nerve-sparing; O (outcome): one or more of the following outcomes: EF, urinary continence and oncologic outcomes; S (study type): cohort studies. Exclusion criteria (1): non-comparative studies; (2) editorial comments, meeting abstracts, case reports, unpublished studies, or reviews; (3) studies with unavailable data for analyses; (4) other studies that did not meet the inclusion criteria.

Data extraction was carried out independently by the two reviewers, which were as follows: (1) general information: the first author extracted the years of publication and countries; (2) population characteristics: the number of patients, age, sample size, criteria to the assess recovery of urinary continence and EF; (3) outcomes: continence recovery (defined as the use of no pad or one safety pad/day), EF recovery (defined as erections sufficient for sexual intercourse), oncologic outcomes: positive surgical margins (PSM). Any discrepancy was resolved through a consensus or consultation with a third reviewer.

### Quality assessment and statistical analysis

The quality of publications was assessed based on the Newcastle-Ottawa Scale (NOS) (https://www.ohri.ca//programs/clinical_epidemiology/oxford.asp); the quality of data extracted from each study was assessed based on case selection, comparability and outcome reporting. A meta-analysis was performed using the Stata 15.1 software (StataSE, USA). Risk ratio (RR) was used as a dichotomous variable. Statistical heterogeneity was evaluated based on *I* ^2^ statistics; *I* ^2^ ≥ 50% indicated a significant heterogeneity, and a random effects model was employed; when *I* ^2^ < 50%, a fixed effects model was employed; p ≤ 0.05 was the threshold for statistical significance (16). Subgroup and sensitivity analyses were performed when it was necessary to explore the sources and sizes of heterogeneity through studies. However, we could not perform sensitivity analyses when comparing three or fewer studies. Publication biases were screened using a funnel plot.

## Results

### Baseline characteristics

**Figure 1:**
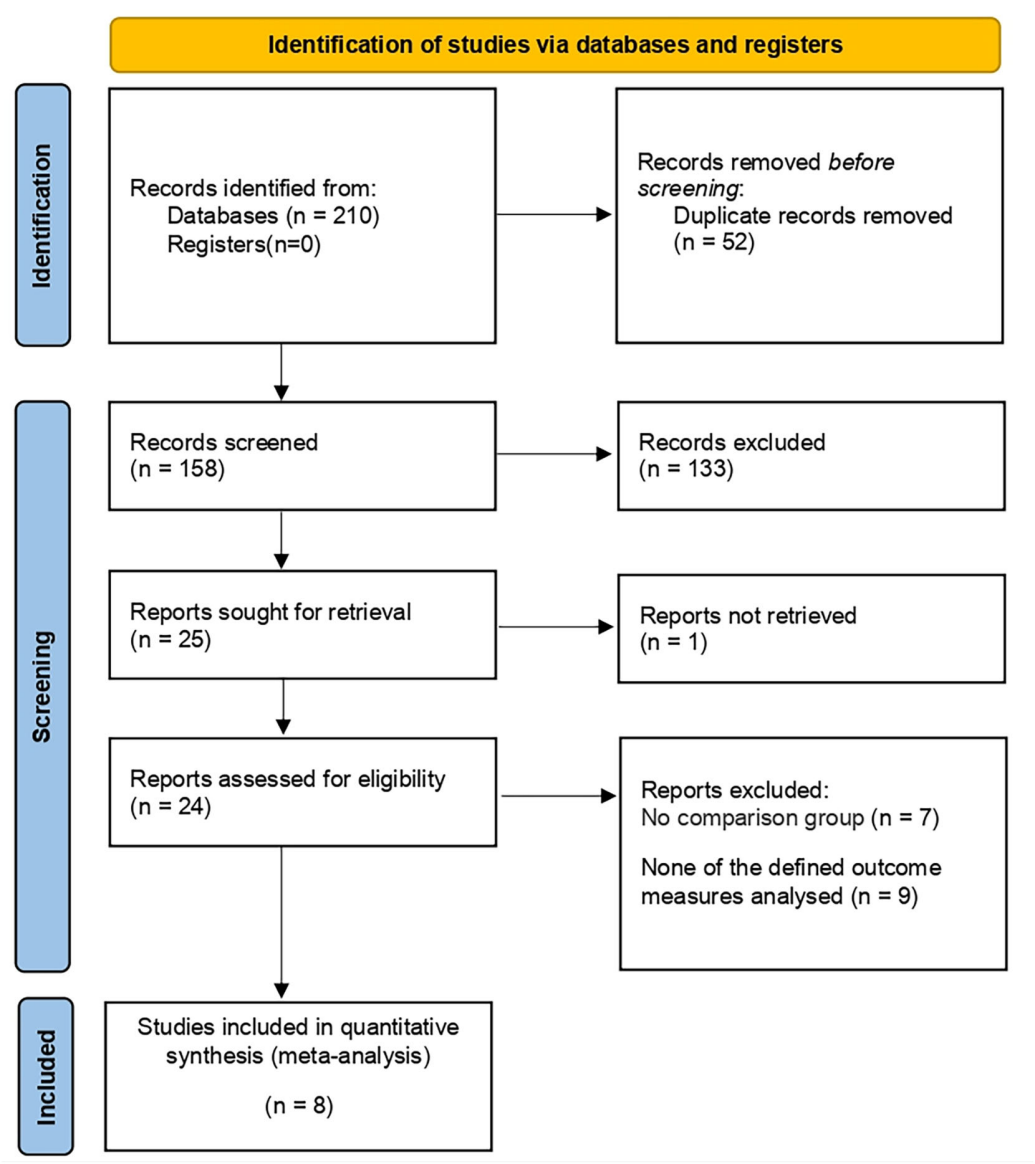
Literature screening flowchart.

210 studies were preliminarily searched, 52 of which remained after duplicates were removed. A total of 24 relevant articles were obtained through the initial screening. We excluded 10 studies after reviewing titles and abstracts, together with 6 articles after reading and screening the full text. 8 prospective or retrospective cohort studies (17–24) were included, ranging from 16 to 769 in size, and the total number of patients included was 2499. The details of the screening processes are shown in **Figure 1**. 90% of the patients in these studies were from European and American countries, and 10% were from Asian countries. The baseline characteristics, the patients included and their associated preoperative variables (age, country, NS type) are summarized in **Table 1**. Due to the different types of NS in some studies (20, 21, 25), we divided it into three types: UNS (partial), BNS and NNS. Furthermore, the definition of urinary continence and the recovery of EF in each study are also included in this table. The preoperative urinary continence and EF of patients in each study, the comparison among studies, the NS standards adopted as well as pathological specimen examinations are summarized in **Table 2**.

**Table 1:** Characteristics of included studies.

Study	Country	Study stype	Sample size	Age, yr	NS type	Outcomes	EF definition	Timing of reported EF	Continence definition	Timing of reported Continence	NOS
Coelho2010	USA	prospective cohort study	144	61(56-66)^a^	U(P)NS/BNS/NNS	PSM	NA	NA	NA	NA	6
Mortezavi2012	Switzerland	retrospective cohort study	16	NA	NS/NNS	PSM	NA	NA	NA	NA	6
Lavery2012	USA	retrospective cohort study	123	NA	U(P)NS/BNS/NNS	PSM	NA	NA	0-1 pad per day	NA	6
Wang2014	China	retrospective cohort study	25	64(57-68)^a^	U(P)NS/BNS/NNS	PSM、EF、Continence	d	12	0-1 pad per day	12	6
Kumar2017	USA	retrospective cohort study	557	BNS:65.1^b^ U(P)NS:61.7^b^ NNS:61.5^b^	U(P)NS/BNS/NNS	PSM、EF、Continence	d	12	0 pad per day	12	5
Abdollah2017	USA	prospective cohort study	769	63(57.5-67)^a^	NS/NNS	EF、Continence	SHIM≥17或d	0、12、24、36	0-1 pad per day	0、12、24、36	7
Takahara2019	Japan	retrospective cohort study	230	NS:64.6 NNS:67.1 ^c^	NS/NNS	PSM	NA	NA	NA	NA	6
Elin2021	Sweden	prospective cohort study	635	NNS:65.8(61.3-68.9) U(P)NS:61.6(56.4-65.3)^a^	U(P)NS/NNS	PSM	NA	NA	NA	NA	7

a median(interquartile).

b Mean(Standard Deviation); BNS, bilateral nerve sparing.

c Mean(range); d, Hardness required for intercourse; EF, Erectile Function; NOS, (Newcastle-Ottawa Scale); NS, nerne-sparing; NNS, non-nerve sparing; SHIM, Sexual Health Inventory for Man; PSM, positive surgical margins; U(P)NS, unilateral (partial) nerve sparing.

**Table 2:** Methodology assessment of included studies.

Study	Baseline continence	Baseline EF	Outcome assessment	Comparability of groups	NS assessment	Pathological specimen examination	Other issues
Coelho2010	NA	NA	NA	Uncontrasted	NA	A positive incisal margin is defined as tumor tissue found on the surface of the ink	Loss of follow up and reason not described
Mortezavi2012	NA	NA	NA	The Gleason score, preoperative clinical stage and positive biopsy number in NNS group were higher than those in NS group	NA	A positive incisal margin is defined as tumor tissue found on the surface of the ink	No lost follow-up
Lavery2012	NA	preoperative SHIM≥16	The doctor or researcher conducted a follow-up SHIM questionnaire	The number of clinical stage and positive biopsy in NNS group was higher than that in NS group	MRI showed ECE, biopsy confirmed SVI. Intraoperative findings and frozen sections	A positive incisal margin is defined as tumor tissue found on the surface of the ink	No lost follow-up
Wang2014	All patients urinary continence normal	SHIM(the second problem≥4)	Questionnaire survey (not specified whether collected by doctors or researchers)	Uncontrasted	NA	NA	No lost follow-up
Kumar2017	NA	No significant difference	Questionnaire conducted by doctor or researcher: SHIM, EPIC, or telephone follow-up	The Gleason score, biopsy positive number and preoperative staging in NNS group were higher than those in NS group	NS : DRE(-) and biopsy positive number < 3; NNS: DRE (+) or positive number ≥4 or ECE suspected	A positive incisal margin is defined as tumor tissue found on the surface of the ink	Loss of follow up and reason not described
Abdollah2017	IPSS:7(3-12)	SHIM:22(15-25)	Questionnaire conducted by physician or researcher: SHIM score and pad use	Uncontrasted	Preoperative functional status of patients, patient willingness, and preoperative assessment of ECE	NA	Loss of follow-up rate of 3.2%; No specific reason for the loss of follow-up was described
Takahara2019	NA	NA	Unspecified urine control monitoring is collected by a doctor or researcher	The mean age, preoperative PSA level, clinical stage and Gleason score in NNS group were higher than those in NS group	BNS : PSA<10ng/ml or cT1c or GS7 or NS positive number <30%; UNS: biopsy positive number <30%; NNS: Beyond the above criteria	NA	No lost follow-up
Elin2021	NA	NA	NA	Preoperative PSA level, clinical stage and pathological stage in NNS group were higher than those in NS group	NA	A positive incisal margin is defined as tumor tissue found on the surface of the ink	Loss of follow up and reason not described

BNS, bilateral nerve sparing; DRE, Digital Rectal Examination; EF, Erectile Function; EPIC, Expanded Prostate cancer Index Composite; ECE, Extra-capsular Extension; IPSS, International Prostate Symptom Score; NS, nerne-sparing; MRI, magnetic resonance imaging; SVI, seminal vesicle invasion; NNS, non-nerve sparing; PSA, prostate-specific antigen; SHIM, Sexual Health Inventory for Man; U(P)NS, unilateral (partial) nerve sparing.

### Assessment of quality

The included studies were scored using the NOS scale. 5 studies were scored 6, 1 study was scored 5, 2 studies were scored 7 and the median NOS of all studies was 6, as is detailed in **Table 3**.

**Table 3:** The risk of bias table.

Study	Selection	Comparability	Outcome	Overall score
Coelho2010	★★★	★	★★	6
Mortezavi2012	★★★	★	★★	6
Lavery2012	★★★	★	★★	6
Wang2014	★★★	★	★★	6
Kumar2017	★★★	★	★	5
Abdollah2017	★★★★	★	★★	7
Takahara2019	★★★	★	★★	6
Elin2021	★★★★	★	★★	7

One ★ represents a score of 1.

### Outcome analysis

#### Urinary continence

**Figure 2:**
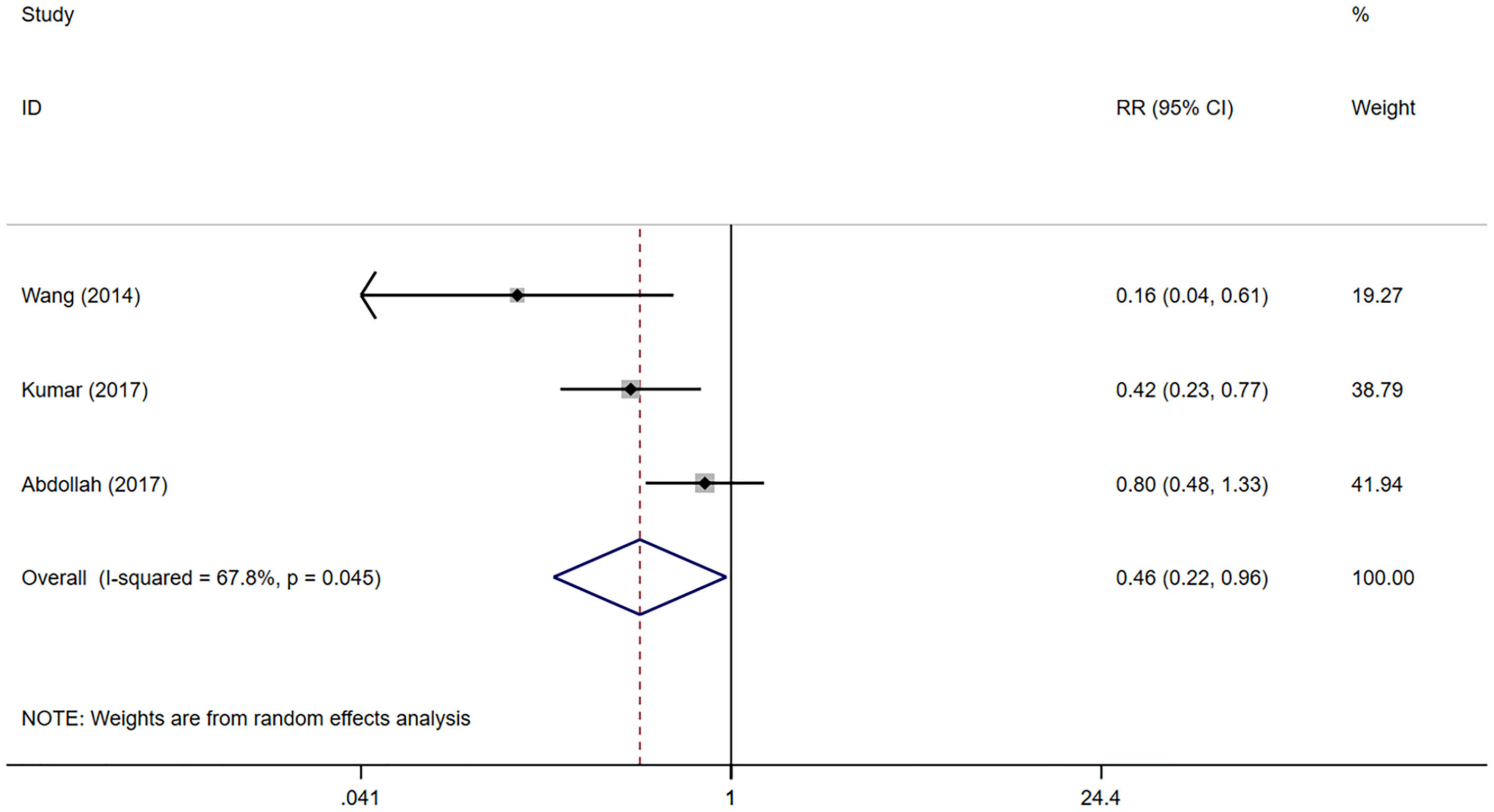
Forest plots of Continence recovery (12 month) for nerve sparing (NS) versus non-nerve sparing. CI, confidence interval; RR, Risk Ratio.

Urinary continence recovery was defined as the use of 0/1 pad per day. The status of urinary continence after surgeries could be extracted from 3 literatures (20–22). Since only the status of urinary continence recovery 12 months after surgeries is reported in the 2 studies (20, 21), we could only conduct a meta-analysis on the data 12 months after surgeries. 1370 patients were included in the 3 studies, including 1220 in the NNS group and 150 in the NS group. The meta-analysis showed that the recovery of urinary control in the NNS group 12 months after surgeries was worse than that in the NS group (RR 0.46, 95%CI 0.22, 0.96; p=0.045 <0.05) (**Figure 2**), and the difference was statistically significant. Due to the small number of studies included, sensitivity analyses could not be performed.

#### Erectile Function

**Figure 3:**
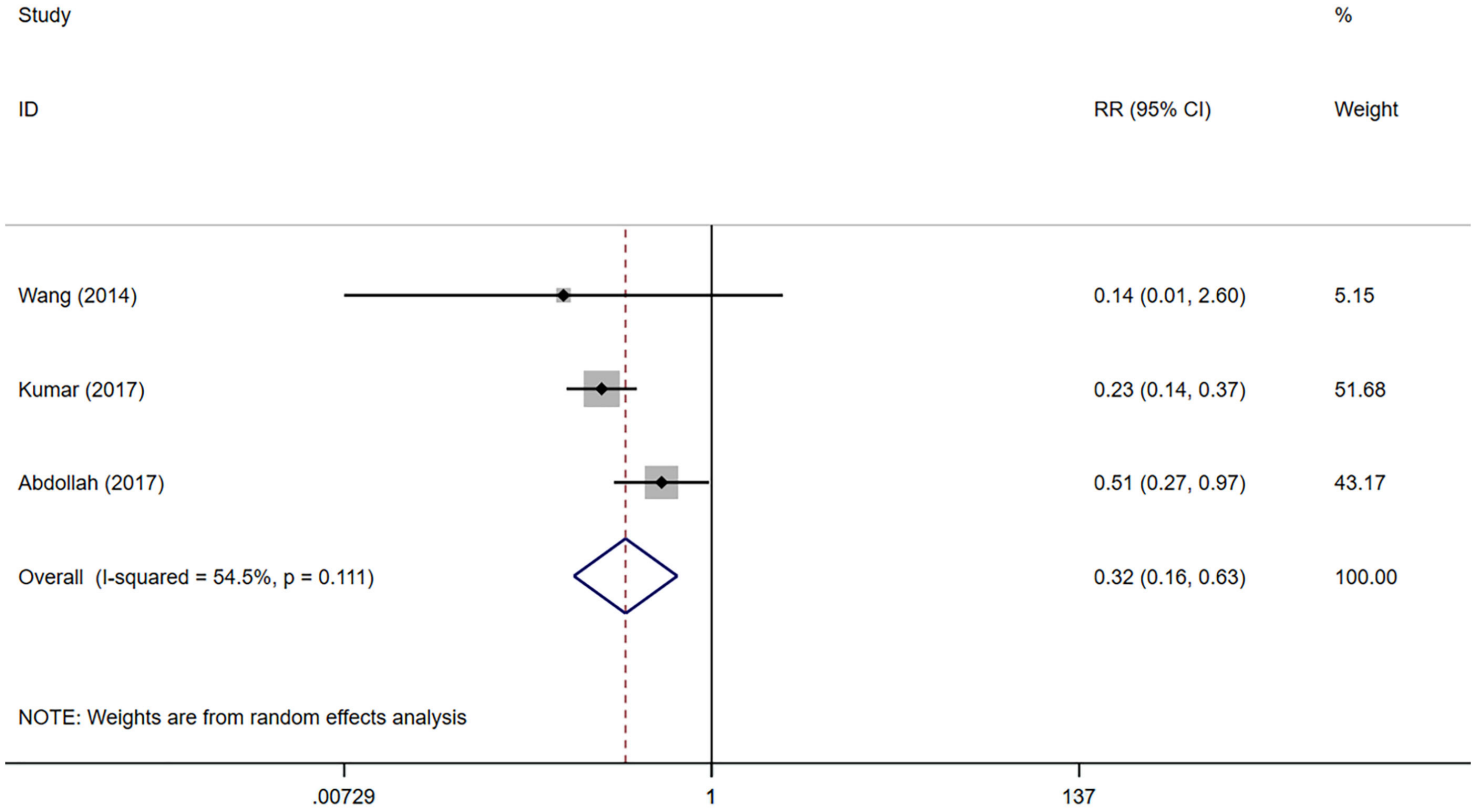
Forest plots of Erectile Function (12 month) for nerve sparing (NS) versus non-nerve sparing. CI, confidence interval; RR, Risk Ratio.

Included in the study, EF was assessed using the SHIM (sexual health inventory for men) scale. EF recovery was defined as SHIM≥17 or an erectile hardness meeting sexual intercourse requirements. The results were evaluated *via* questionnaires or telephone follow-ups. EF recovery data was able to be extracted from 3 studies (20–22). However, only the recovery of EF 12 months after surgeries is reported in the 2 studies (20, 21), so we could only conduct a meta-analysis on the recovery of EF 12 months after surgeries. 1130 patients were included in the 3 studies, including 633 in the NNS group and 497 in the NS group. The results of meta-analysis showed that the EF recovery was worse in the NNS group than in the NS group 12 months after surgeries (RR 0.32, 95%CI 0.16, 0.63; p=0.001 <0.05) (**Figure 3**), and the difference was statistically significant. Due to the small number of studies included, sensitivity analyses could not be performed.

### Oncologic outcomes

**Figure 4:**
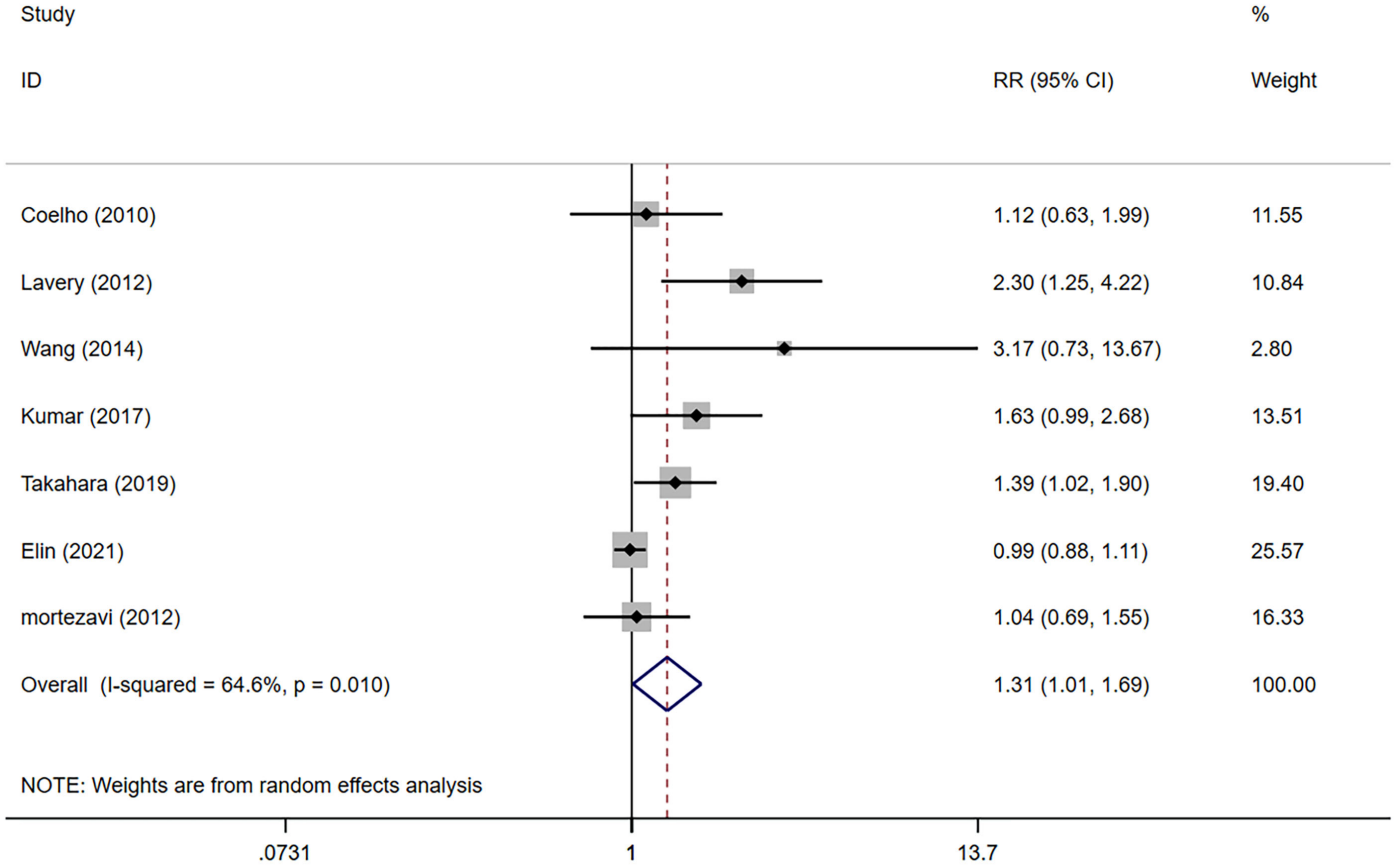
Forest plots of Oncologic Outcomes for non-nerve sparing versus nerve sparing (NS). CI, confidence interval; RR, Risk Ratio.

The meta-analysis showed that the NSS group presented higher positive surgical margin (PSM) rates compared with the NS group (RR 1.31, 95% CI 1.01, 1.69; p=0.042 <0.05) (**Figure 4**), and the difference was statistically significant.

### Subgroup analysis

**Figure 5:**
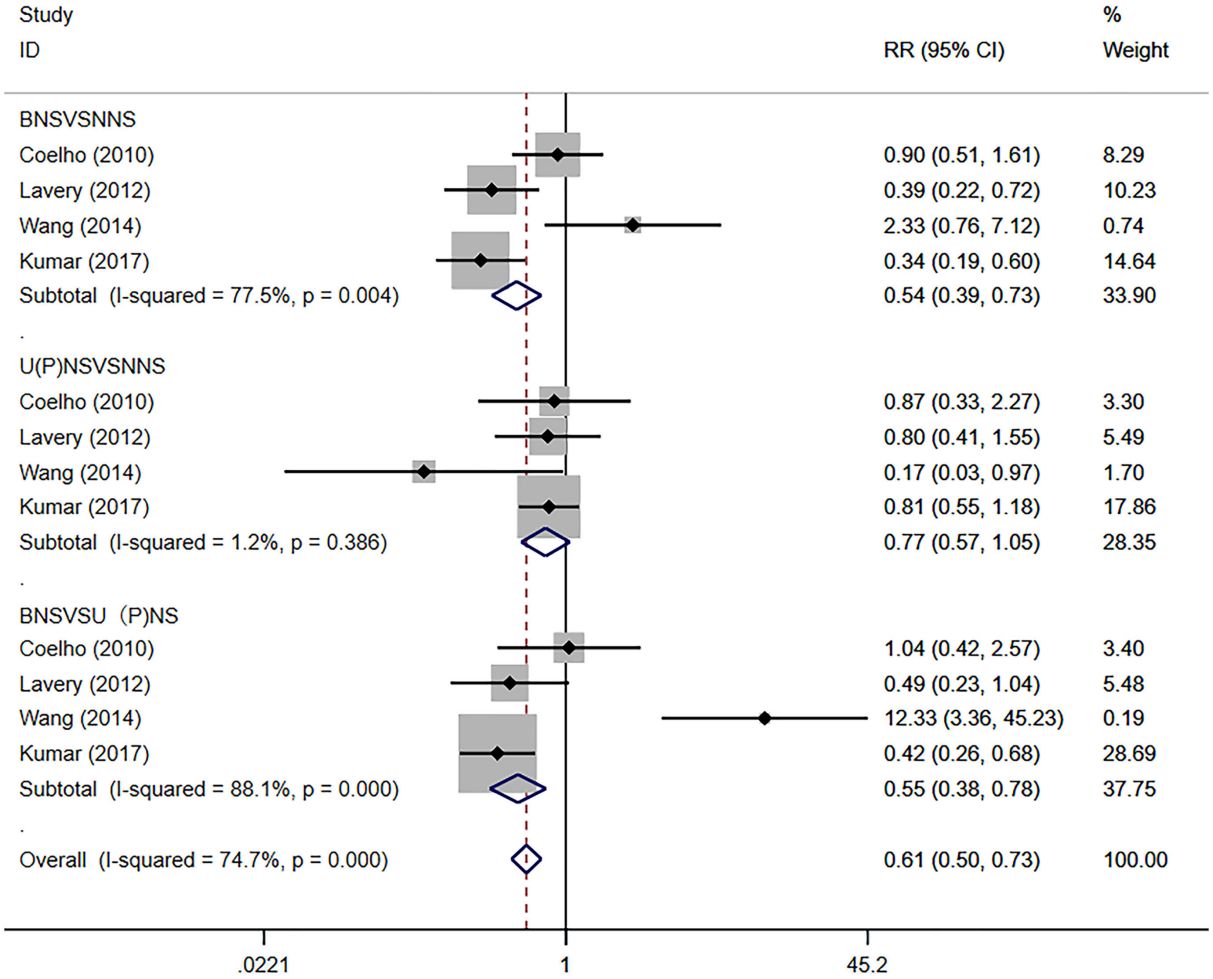
Forest plots of PSM Subgroup analysis for nerve sparing (NS) versus non-nerve sparing. CI, confidence interval; RR, Risk Ratio; PSM, positive surgical margins.

We performed a meta-analysis on PSM according to the type of NS. The results showed that the BNS group presented lower PSM rates compared with the NNS group (RR 0.54, 95% CI 0.39, 0.73; p=0.004 <0.05). There was no significant difference in the PSM of the NNS group and the U(P)NS group (RR 0.77, 95% CI 0.57, 1.05; p>0.05). The BNS group showed lower PSM rates compared with the U(P)NS group (RR 0.55, 95% CI 0.38, 0.78; p=0.001 <0.05) (**Figure 5**).

### Publication bias and sensitivity analysis

**Figure 6:**
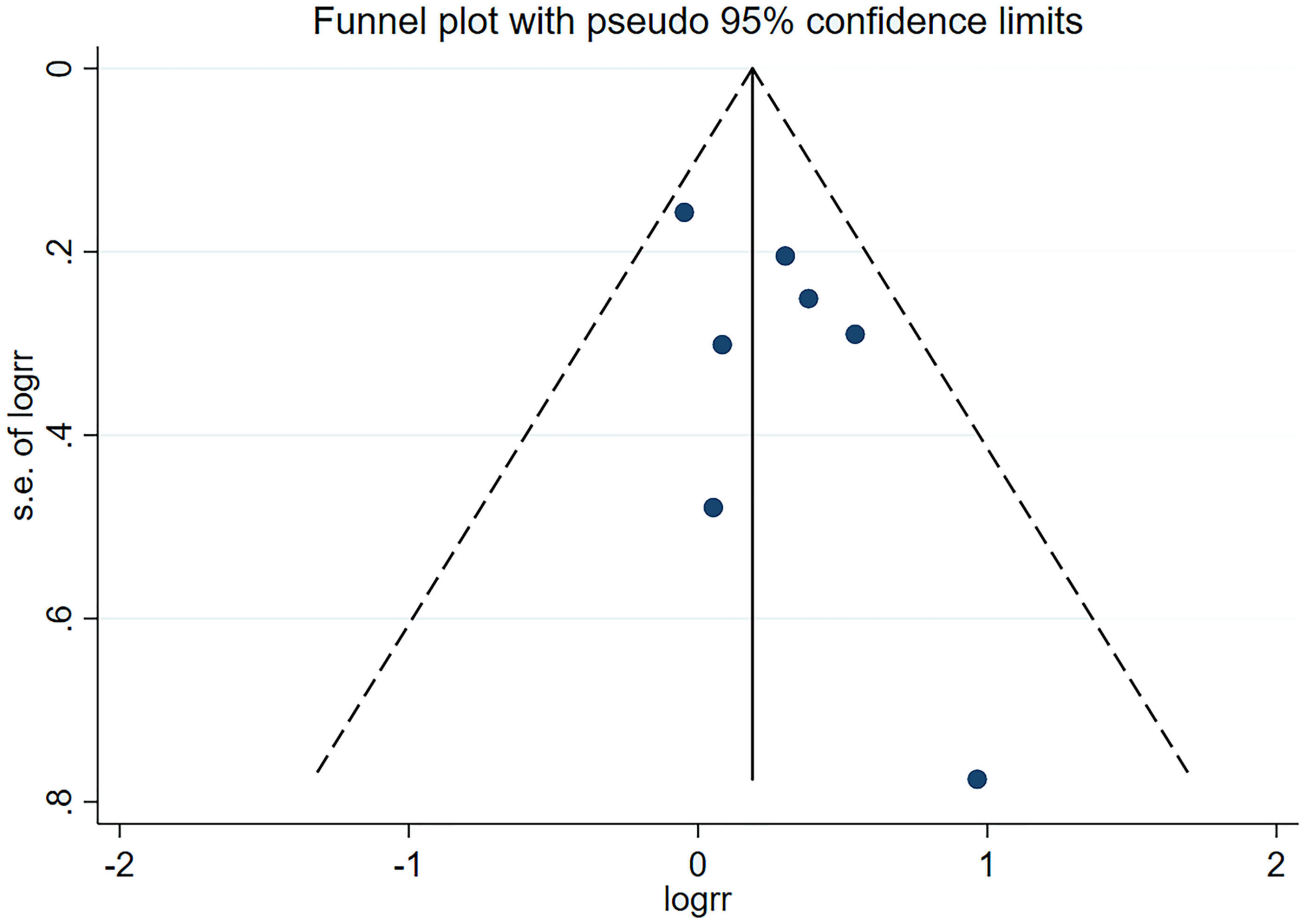
Funnel plot of positive surgical margins for publication bias.

The result showed no apparent asymmetry, which indicated no obvious publication bias (**Figure 6**), and sensitivity analyses revealed that no significant influence was produced on the results through the removal of any article.

## Discussion

Some significant findings on the urinary continence, EF and oncologic outcomes of the present study warrant in-depth discussions.

### Urinary continence

Kumar and Wang et al. defined the criteria for the recovery of urinary continence as: 0 pad per day; Abdollah, Lavery et al. defined it as: 0/1 pad per day. It was found through a meta-analysis on the recovery of urinary continence 12 months after surgeries in the NNS group and the NS group that intraoperative NS was beneficial to the recovery of urinary continence 12 months after surgeries for high-risk Pca. Faieleigh et al. (13) conducted a systematic evaluation on the effect of NS during RP on postoperative urinary continence in 2015. The results showed that NS could improve the results of urinary continence recovery 6 months before and after surgeries, but there was no significant improvement in urinary continence 12 months after surgeries, which seemed different from our study. This might be due to the focus on anterior reconstruction through periurethral suspension suture as well as the posterior reconstruction *via* reattachment of the tendon arch (26) and pubic anterior plate to the bladder neck (26) through RARP compared to traditional laparoscopic radical prostatectomy (LRP). Although these techniques appear to have a similar efficacy to LRP 1 or 3 months after surgeries, there is a statistically-significant difference 12 months after surgeries (27). At present, the pathophysiological process of urinary incontinence after RP is not clear, and the function or structure of the striated sphincter may be the cause of urinary incontinence (28–30). The urethral sphincter is divided into two mechanisms: the striated sphincter provides active urine control under stress, and the smooth sphincter provides passive urine control (31). The two types of smooth muscles have different innervations. The smooth sphincter mainly receives autonomic innervations from the pelvic plexus, while the striated sphincter is innervated by dual nerves, namely the pelvic nerve, the pelvic branch of the pudendal nerve (32) and the abnormal innervations of the sphincter from the somatic nerve in the pelvic cavity (32–34). These studies may explain the effect of NS on postoperative urinary continence. Pelvic lymphadenectomy (LAE) is a routine procedure in the treatment of patients with high-risk Pca through RARP, which may damage the nerve fibers adjacent to the pelvic plexus. Therefore, careful attention should be paid to LAE during NS surgeries to avoid the possibility of nerve damage (35). Martin et al. summarized the current surgical approaches and anatomical techniques of RARP, which provide us with more options for surgical approaches (36). In addition, it is mentioned in the study of kumar et al. (21) that pelvic floor muscle exercise after surgeries, which can improve patients’ urine continence function recovery after surgeries (37). Unfortunately, we don’t know the statistics or follow-ups of the study, so we don’t know whether it is a contributing factor.

### Erectile function

The recovery of sexual function in almost all studies has been “with or without the use of drugs, hardness is required for intercourse”. Our study shows that NS during RARP can promote EF recovery for patients with high-risk prostate 12 months after surgeries. The recovery of EF after surgeries was not entirely determined through NS. Holze et al. (38) demonstrated the potential effect of NS techniques, preoperative EF and pelvic LAE on the postoperative recovery of EF. At the same time, KIM et al. (39) also emphasized that a higher serum testosterone level before surgeries was also a factor influencing the postoperative recovery of EF. Abdollah et al. (22) also pointed out that the type of NS was largely determined by the recovery of EF. The EF recovery rate 12, 24 and 36 months after NNS was 19.6%, 19.6% and 39.7% respectively, compared with 42.1%, 67.1% and 75.3% after BNS as well as 31.2%, 47.3% and 69.5% after U (P) NS. Novara et al. (40) believed that the best effect of NS could be achieved by patients with a good EF at baseline. In other words, patients with a decreased EF before surgeries should not be treated with NS during surgeries. Combined with current studies, we believe that RARP in the treatment of high-risk Pca through NS is beneficial to the recovery of sexual dysfunction 12 months after surgeries.

### Oncologic outcomes

In most studies, “tumor tissues found on the surface of ink” are taken as the definition and standard of positive surgical margins. Some studies have shown that PSM is equivalent to residual tumors as an adverse surgical outcome significantly associated with postoperative recurrence and secondary treatment (41, 42). Our meta-analysis showed that the PSM rate could increase through intraoperative NNS, a subgroup analysis showed that the PSM rate in the BNS group was lower than that in the U (P) NS group, and the difference was statistically significant. This seems to differ from the findings of Veneziano et al. (14). Lavery et al. (19) found through a comparison among study groups that patients in the NS group had lower pathological scores, showed fewer tumors, invaded extracellular membranes and seminal vesicle glands, and a higher proportion of tumor stages was T2. Kumar (21) also pointed out that the NNS group was significantly higher than the NS group in tumor invasion rate, pathological stage and tumor volume, and that there was a selection bias. The univariate and multivariate regression analysis in their study showed that NS did not affect PSM (p=0.341). These oncolologic features will be considered by the surgeons for NS treatment. In addition, PSM is also related to the age, PSA level, tumor stage (43), surgical experience (44) and tumor conformity of high-risk Pca itself. Our study confirmed that through NS in RARP, the rate of PSM can be reduced, and that the higher the degree of preservation was, the lower the risks would be. This result needs to be treated with caution.

Through a study of Kumar, Lavery and Takahara et al., we found that the mean age, biopsy Gleason score, preoperative clinical stage and preoperative PSA level of the NS group were significantly lower than those of the NNS group, suggesting that the patients included in our study had a selection bias, which might be solved through preoperative randomization, but it might violate ethics. In addition, we did not specifically group the methods of intraoperative NS. In studies on PNS (21, 24) intrafascial and interfascial PNS was also included. Again, we cannot address the effect of these classifications on the results.

Some limitations of our study should be considered before interpreting our results. First of all, all the literatures we included were cohort studies, which failed to include randomly-controlled trials with higher levels of evidence, although they were qualified by the NOS scale. The studies included involved a retrospective design and all risks of bias. Even through modern statistics methods, some issues could not be addressed (selection bias, nerve-sparing degree, postoperative protocols, etc.). Secondly, due to limited data, the situation of 1, 3, 6 and 24 months after surgeries was not further discussed. In addition, although a comparison of baseline data between the 2 treatment groups was described in the 3 studies included, fewer details were described. Therefore, it is unknown whether our interpretation of results would be affected. We expect that in follow-up studies, more attention will be paid to this point. Moreover, we have no consensus on the classification of NS, nor is there a standard classification of NS at present. Postoperative outcomes may be different due to different types of NS, and the result depends on its percentage (45, 46). There is heterogeneity in the NS techniques, which is not addressed in most studies. The authors performed different grades of NS (Kumar and Elin, etc.), which could influence its percentage. Finally, the postoperative rehabilitation protocols for potency and continence were unclear in the studies included, which could also be an issue when assessing postoperative results.

Due to the differences in postoperative follow-ups, surgical techniques and medical levels in different regions among the studies included, as well as the inevitable selective bias in the NSS group in terms of oncology characteristics. Our results need to be carefully interpreted and verified through high-quality studies.

## Conclusions

NS is feasible during RARP surgeries, which is beneficial for the long-term (12 months after surgeries) urination continence and EF recovery of patients with high-risk Pca and is associated with better oncological outcomes. It is encouraging to find that NS may significantly improve the life quality of patients high-risk Pca after surgeries, which thus actively cooperates with treatments. However, due to interference factors in studies, it is not clear whether this advantage is caused by NS, and clinical decisions should be combined with various considerations on whether to perform NS.

## Data availability statement

The original contributions presented in the study are included in the article/supplementary material. Further inquiries can be directed to the corresponding author.

## Author contributions

YL, X-ZD, JQ and X-SY: Protocol development, data collection and management, data analysis and manuscript writing. ZW, YJ and JH: Data management, data analysis and manuscript writing, data management, data analysis and manuscript writing. C-JW, LW, K-PL and J-HW: Data management and manuscript writing. All authors contributed to the article and approved the submitted version.
